# Topological analysis of functional connectivity in Parkinson’s disease

**DOI:** 10.3389/fnins.2023.1236128

**Published:** 2023-08-23

**Authors:** Weiwei Zhang, Shengxiang Xia, Xinhua Tang, Xianfu Zhang, Di Liang, Yinuo Wang

**Affiliations:** ^1^School of Science, Shandong Jianzhu University, Jinan, China; ^2^School of Cyberspace Security, Shandong University of Political Science and Law, Jinan, China; ^3^School of Control Science and Engineering, Shandong University, Jinan, China; ^4^School of Nursing and Rehabilitation, Cheeloo College of Medicine, Shandong University, Jinan, China

**Keywords:** resting-state fMRI, Parkinson’s disease, persistent homology, functional connectivity neural circuits, VR complex

## Abstract

Parkinson’s disease (PD) is a clinically heterogeneous disorder, which mainly affects patients’ motor and non-motor function. Functional connectivity was preliminary explored and studied through resting state functional magnetic resonance imaging (rsfMRI). Through the topological analysis of 54 PD scans and 31 age-matched normal controls (NC) in the Neurocon dataset, leveraging on rsfMRI data, the brain functional connection and the Vietoris-Rips (VR) complex were constructed. The barcodes of the complex were calculated to reflect the changes of functional connectivity neural circuits (FCNC) in brain network. The 0-dimensional Betti number *β_0_* means the number of connected branches in VR complex. The average number of connected branches in PD group was greater than that in NC group when the threshold *δ* ≤ 0.7. Two-sample Mann–Whitney *U* test and false discovery rate (FDR) correction were used for statistical analysis to investigate the FCNC changes between PD and NC groups. In PD group, under threshold of 0.7, the number of FCNC involved was significantly differences and these brain regions include the Cuneus_R, Lingual_R, Fusiform_R and Heschl_R. There are also significant differences in brain regions in the Frontal_Inf_Orb_R and Pallidum_R, when the threshold increased to 0.8 and 0.9 (*p* < 0.05). In addition, when the length of FCNC was medium, there was a significant statistical difference between the PD group and the NC group in the Neurocon dataset and the Parkinson’s Progression Markers Initiative (PPMI) dataset. Topological analysis based on rsfMRI data may provide comprehensive information about the changes of FCNC and may provide an alternative for clinical differential diagnosis.

## 1. Introduction

Parkinson’s disease (PD) is a clinically degenerative disorder disease of the nervous system with motor and non-motor symptoms. The disease mainly affects people’s motor function, such as bradykinesia, tremor, muscle stiffness or rigidity, abnormal walking gait, etc. In addition, it will also affect non-motor function, such as cognitive impairment, insomnia, depression, autonomic nerve dysfunction, and so on. The cause of PD is unclear. In the early premotor stages, the diagnosis of this disease is still difficult (Aarsland et al., 2017). Anatomical magnetic resonance imaging could not detect the loss of dopamine neurons. With the development of molecular biology, neural structure, and functional imaging technology, more and more biomarkers of PD can be discovered, providing the possibility for early diagnosis, disease monitoring, and differential diagnosis, thus achieving accurate early intervention and efficacy evaluation of the disease. Dopamine transporter single photon emission computed tomography (DAT-SPECT) and resting state functional magnetic resonance imaging (rsfMRI) are potential techniques for detecting the survival status of neurons in PD (Wang et al., 2012). Among them, DAT-SPECT can provide quantitative information about dopamine neurons, which is very useful for assessing disease severity and monitoring treatment effectiveness. However, it is expensive and carries the risk of radioactive tracer. On the other hand, rsfMRI is a non-invasive imaging technique that is relatively inexpensive and can avoid ionizing radiation. It can provide information about brain activity, which is very useful for studying the neural mechanisms of PD and evaluating treatment effectiveness.

Therefore, the study of functional connectivity (FC) based on rsfMRI is a promising method. Based on the correlation of time series, FC was preliminary explored and studied through rsfMRI (Aarsland et al., 2017). With rsfMRI, FC can be used to detect a variety of diseases such as Alzheimer’s disease, PD, schizophrenia, and so on (Lee et al., 2013). Some studies have shown that motor and cognitive impairment in PD are related to abnormal functional connections (Putcha et al., 2015; Zhang et al., 2015). Graph theory-based approaches used in PD research have shown that topological properties of brain networks are disrupted, which can help identify this type of disease (Luo et al., 2015). Specifically, abnormal local and global network efficiency changes suggest clinical phenomena in PD. The above methods assume that the functional network is stable and does not change over time. A dynamic FC based method was used in recent research. For example, previous studies used rsfMRI and sliding windows to assess differences in dynamic connectivity between normal control (NC) and PD (Kim et al., 2017). Huang et al. (2020) proposed a regression method to model the dynamic correlation matrices as a linear combination of symmetric positive definite matrix to smooth the image acquisition and physiological noise. Pang et al. (2021) distinguished different motor subtypes of PD based on multilevel indices of rsfMRI and Support Vector Machine (SVM).

However, the graph theory analysis method can not describe the characteristics of higher-level complex brain networks. To study the topological characteristics of complex brain networks on a larger scale, researchers began to study Vietoris-Rips (VR) complex filtration based on persistent homology in brain networks. In topological data analysis (TDA), persistent homology is an effective tool to explore the nonlinear structure of the data. Compared with the common methods such as principal component analysis (PCA), cluster analysis, and graph theory (Li et al., 2009), TDA can effectively capture the topological information of high-dimensional data space. This kind of algorithm adopts a free threshold and solves the problem of threshold selection. It measures the topological characteristics of brain network under all possible thresholds (Choi et al., 2014; Chung et al., 2015; Lee et al., 2017). These approaches mainly associate the 0-dimensional Betti numbers *β_0_* with current varying thresholds. A connected component-based aggregation cost model called Integrated Persistence Features (IPF) was proposed in previous research (Kuang et al., 2019). Different from the above persistent homology feature which based on 0-dimensional Betti numbers, this paper proposes a persistent homology feature based on the 1-dimensional Betti number β_1_. To our knowledge, there is little literature investigating the 1-dimensional Betti number β_1_ in PD. Our main contributions of this paper are as follows: (1) The VR complex filtering model was established based on the relationship matrix of the human brain network. The persistent homology method was used to calculate VR filtered barcodes. And then the functional connectivity neural circuits (FCNC) at different thresholds are calculated from the barcodes. (2) Two-sample Mann–Whitney *U* test and FDR correction are used for statistical analysis to investigate the FCNC changes between PD and NC groups. (3) Through the statistical tests on the number of FCNC in PD and NC groups, our results show that there is a significant statistical difference between the PD group and the NC group.

## 2. Method

### 2.1. Basic concepts about persistent homology

The common method to reduce the dimension of data is PCA, but this method will lose some potentially valuable data more or less. Persistent homology provides us a method to find a complete data ship without dimensional reduction.

Persistent homology is an effective tool to analyze high-dimensional data and explore the nonlinear structure of data. It can calculate topological features at different spatial resolutions. By identifying persistent topological features over changing scales, persistent homology provides clues for effective analysis of multi-scale networks. Its core idea is to analyze the birth and death of holes in various dimensions in a multi-scale range. To extract persistent homology features, we first need to construct VR complex. Let d(·, ·) denote the distance between two points in the metric space Z. The value of δ denotes the threshold. When we change the threshold, we obtain a sequence of complexes. The VR(Z, δ) complex is defined as follows (Silva and Carlsson, 2004):

1. For vertices *a* and *b*, edge [*ab*] is included in VR(*Z*, δ) if *d*(*a,b*) ≤ δ.
2. A higher dimensional simplex is included in VR(*Z*, δ) if all of its edges are included in it.

Note that VR(Z, δ_0_) ⊆ VR(Z, δ_1_) ⊆ · · · ⊆ VR(Z, δ_n_), for δ_0_ ≤ δ_1_ ≤ · · · ≤ δn. Therefore, the VR complex VR(X, δ_i_) (*i* = *0, 1, · · ·, n*) is a filtered simplicial complex.

Betti intervals help describe how the homology of VR(Z, δ) changes with δ. A k-dimensional Betti interval, with endpoints (δ_start_, δ_end_), corresponds roughly to a k-dimensional hole that appears at the threshold δ_start_, remains open for δ_start_ ≤ δ < δ_end_, and closes at δ_end_.

The rank of the homology group is called Betti number (Woo et al., 2014), which is a set of important topological invariants. It uses the connectivity based on k-dimensional simplex complex to distinguish the topological space, which can well reflect the topological structure of an object. The k-dimensional Betti number is the rank of the k-th homology group and represents the number of “holes” in the *k*-th dimension. For example, the 0-dimensional Betti number β_0_ refers to the number of connected branches (Lee et al., 2012). Similarly, the one-dimensional Betti number β_1_ intuitively represents the number of one-dimensional “holes.”

### 2.2. An example of persistent homology

**Figure 1:**
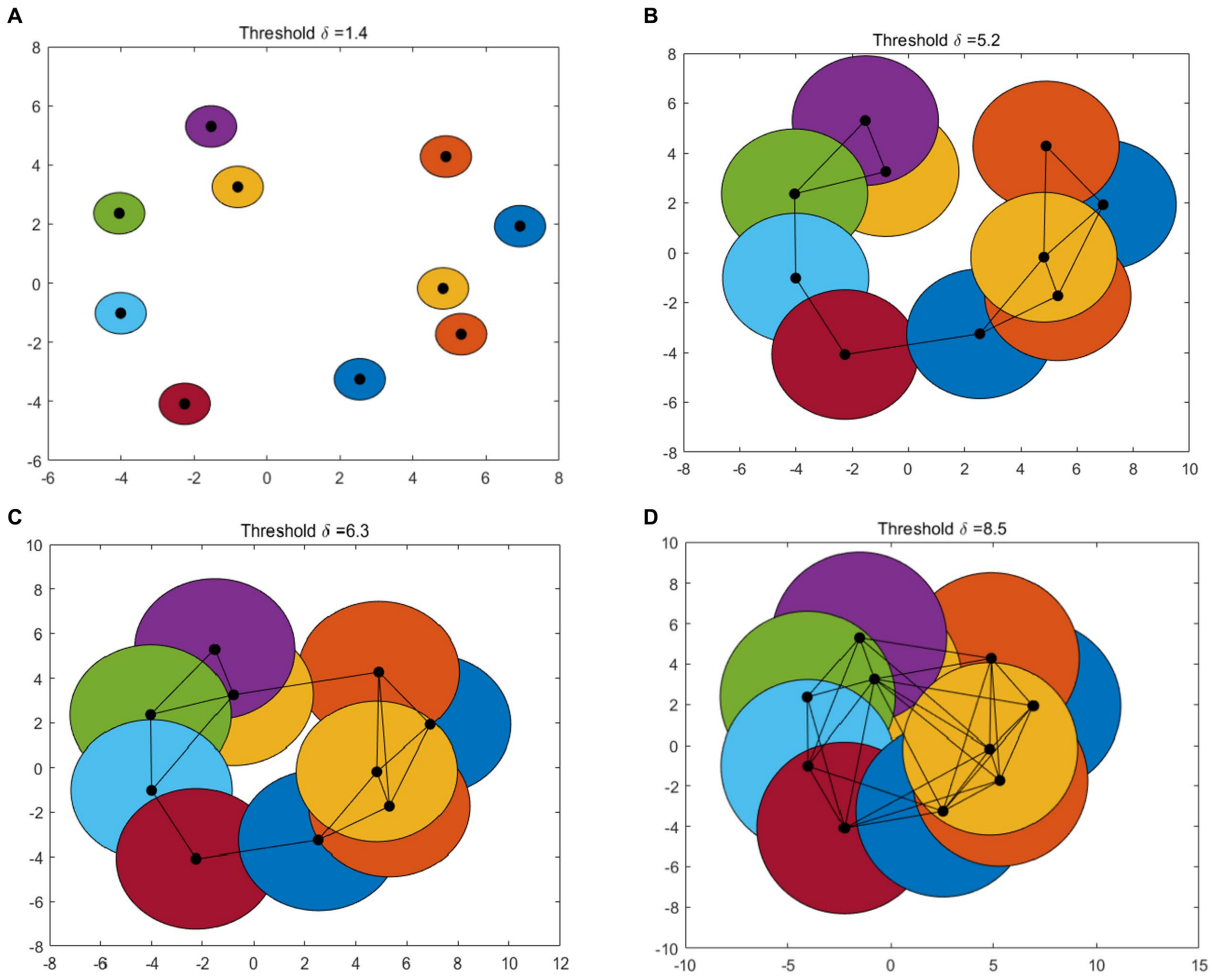
An example of VR complex. Betti number changes with **(A–D)**: *δ* = 1.4, 5.2, 6.3, and 8.5, respectively.

Figure 1 gives an example of Betti number changes with *δ* = 1.4, 5.2, 6.3, and 8.5, respectively. Take 10 points randomly and draw a circle centered on these points and radius of the threshold δ/2. In the process of increasing the threshold δ from 0 to the maximum, the 0-dimensional and 1-dimensional Betti numbers change constantly. When *δ* = 1.4, the number of connected branches is 10, that means *β*_0_ = 10 and the number of 1-dimensional “holes” *β*_1_ = 0 (Figure 1A). In Figure 1B, when *δ* = 5.2, β_0_ = 1 and β_1_ = 0. As the threshold δ increase, when *δ* = 6.3 (Figure 1C), *β*_0_ = 1 and *β*_1_ = 1. When *δ* = 8.5, some holes are “filled.” At this time, there is only one connected branches (*β*_0_ = 1) and one 1-dimensional “holes” (*β*_1_ = 0) (Figure 1D).

**Figure 2:**
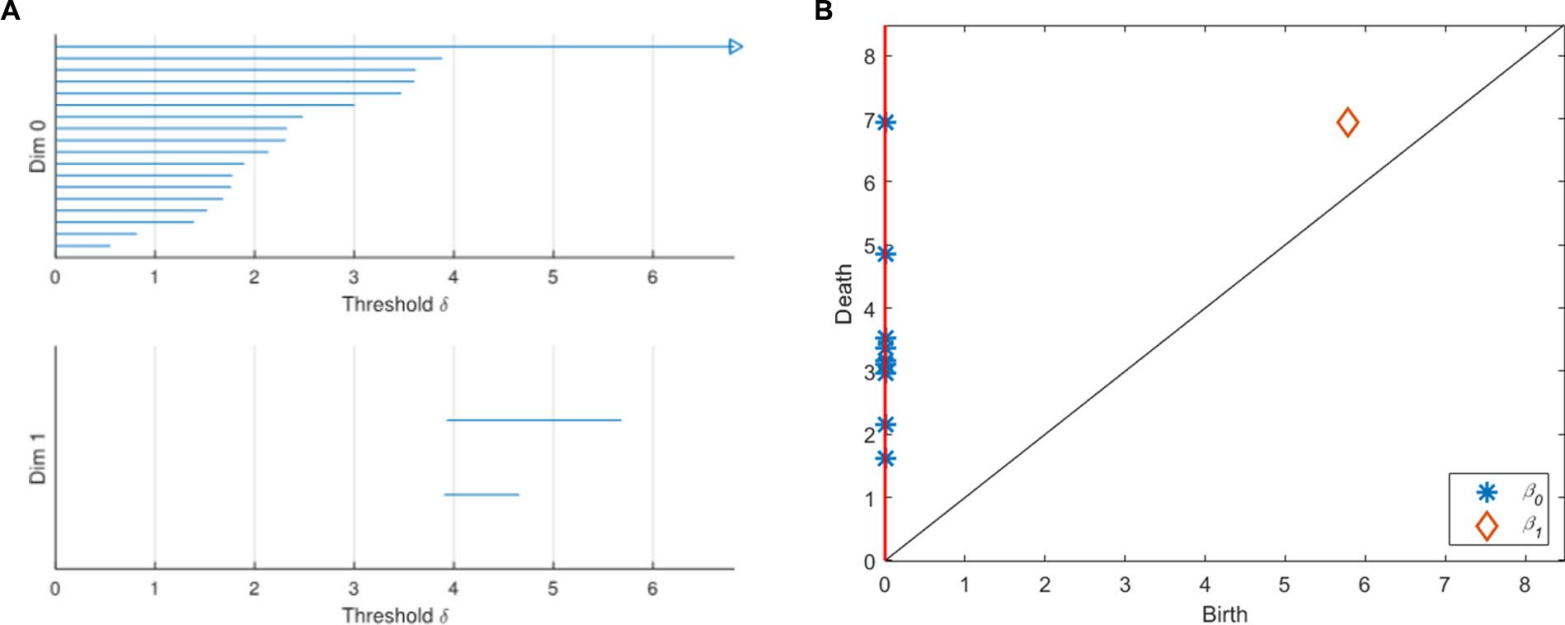
Barcodes and persistence graph. **(A)** Barcodes. **(B)** Persistence diagram.

With the change of the threshold δ, the topological characteristics of VR complex change. This process can be represented by a barcode and a persistence diagram (Figure 2). As shown in Figure 2A, a barcode is a set of finite intervals. Each interval represents the birth and death of holes in the corresponding dimension, and these intervals are parallel to each other. K-dimensional barcodes (*k* = 0, 1 in Figure 2A) show us the duration of k-dimensional topological features. Generally, we regard the features with very short duration as noise, and the features with long duration as real signal features. In Figure 2B, the persistence diagram provides a multi-scale feature description. The abscissa of each point in the diagram represents the birth of the topological feature, while the ordinate represents its demise. Points away from the diagonal represent features with a long life cycle, while points close to the diagonal represent features with a short life cycle. Among them, the feature that can be maintained for a longer time is a useful persistence feature with stronger robustness. The feature with short life is more likely to be noise or detail. The red vertical line on the ordinate axis is regarded as a point at infinity, representing a topological feature that will never die.

## 3. Results

### 3.1. Dataset and preprocessing

The datasets used in this article are the Neurocon dataset (Badea et al., 2017) and Parkinson’s Progression Markers Initiative (PPMI) dataset (Marek et al., 2011). The Neurocon dataset includes rsfMRI data from 27 PD patients and 16 age matched NC patients, with each subject undergoing 2 repeated scans. One NC scan was subsequently excluded owing to data corruption. Finally, 54 PD scans and 31 NC scans were included in the final analyses. The Neurocon study has been approved by the Ethics Committee of the Emergency Hospital of the University of Bucharest and is in line with the Helsinki Declaration 1964 and its later revised ethical standards (Badea et al., 2017). The scanner is Siemens AVanto 1.5 T, Scan time = 8.05 min, TR = 3.48 s, Voxel = 3.8 × 3.8 × 5 mm^3^, volume = 137. Each rsfMRI consists of 2 mm isotropic voxels and 1,200 time points over a 14 min, 33 s scanning session. The details of the Neurocon dataset are shown in Table 1. Retrieve rsfMRI data from the PPMI dataset for 154 PD patients and 24 age matched NC patients. Each study in the PPMI dataset was approved by the Human Experimental Ethics Standards Committee before registration, and each subject signed a written informed consent form. This study obtained the right to use the PPMI database data.

**Table 1:** The details of the Neurocon dataset.

Dataset	PD subjects	NC subjects	PD scans	NC scans	Age PD (mean ± SD)	Age NC (mean ± SD)	P (age NC-PD)	H&Y (mean ± SD)	Disease duration (mean ± SD)
Neurocon	27 (16 M)	16 (5 M)	54	31	68.7 ± 10.6	67.6 ± 11.9	0.76	1.92 ± 0.33	4.6 ± 6.5

SD, standard deviation; H&Y, Hoehn and Yahr; PD, Parkinson’s disease; NC, normal controls.

The Neurocon dataset is preprocessed using DPABI (DPABI Software Library v5.1) (Yan et al., 2016) as follows:

1. Removing the first 10 time points of rsfMRI data to obtain a stable signal;
2. Time correction is performed on each slice to ensure that the data on each slice corresponds to the same time;
3. Realign: eliminate the data with the maximum value of translation greater than 3 mm and the maximum value of rotation greater than 3^°^;
4. Registration of structural image to functional image space. Use anatomical T1 images to register to the standard Montreal Institute of Neurology MNI152 template;
5. Check the coverage of function image by Automask;
6. Bandpass filtering with a frequency of 0.01 ~ 0.1 Hz is set;
7. Normalize using EPI templates;
8. Extract region of interest (ROI) time courses using Anatomical Automatic Labeling (AAL) atlas.

The brain parcellation used in this paper is the AAL brain template (Tzourio-Mazoyer et al., 2002). There are 116 regions in the AAL template, 90 belonging to the brain, and the remaining 26 belong to the cerebellar structure. There are few studies on the cerebellum. In this paper, the classical AAL template of 90 brain regions is used to construct VR complex.

### 3.2. Constructing VR complex

Firstly, we preprocess the rsfMRI image as described above, and then calculate the FC between the 90 brain regions in the AAL template. Pearson correlation is probably the most commonly used scheme for calculating functional connections (Valdes-Sosa et al., 2011). We also use the Pearson correlation coefficient between vertices to construct the FC matrix in this work. FC is defined as the temporal correlation of brain region. For convenience, we defined *P*(j) = {*P*_1_(j), *P*_2_(j), · · ·, *P*_n_(j)} (*j* = 1, 2, · · ·, 90) as the average time signal sequence of the *j*-th brain regions, and n is the total number of time series. The FC matrix M = (*m*_ij_) (*i, j* = 1, 2, · · ·, 90) is calculated using equation (1):


(1) $$m_{ij}=corr(P^{i},P^{j})=\frac{\sum_{k=1}^{n}\left(P_{k}^{\left(i\right)}-{\bar{P}}^{\left(i\right)})(P_{k}^{\left(j\right)}-{\bar{P}}^{\left(j\right)}\right)}{\sqrt{\sum_{k=1}^{n}{\left(P_{k}^{\left(i\right)}-{\bar{P}}^{\left(i\right)}\right)}^{2}}\sqrt{\sum_{k=1}^{n}{\left(P_{k}^{\left(j\right)}-{\bar{P}}^{\left(j\right)}\right)}^{2}}}$$

To construct VR complex, we defined the distance between each two vertices using equation (2):


(2) $$d\left(P^{i},P^{j}\right)=\sqrt{1-corr\left(P^{i},P^{j}\right)}\;\;\left(i,j=1,2,\cdots ,90\right)$$

For each value, the nested complex $VR\left(Z,\delta_{0}\right)\subseteq VR\left(Z,\delta_{1}\right)\subseteq \cdots \subseteq VR\left(Z,\delta_{n}\right)$ is constructed. Thus, a VR complex was further established for each subject. The VR complex take three inputs: the maximum dimension d_max_ of any included simplex, the maximum filtration value t_max_, and the number of divisions. In our experiments, the maximum dimension d_max_ = 2, which means we construct 0-dimensional simplex and 1-dimensional simplex. The maximum filtration value t_max_ = 0.9 and the number of division is set to 450.

## 4. Discussion

**Figure 3:**
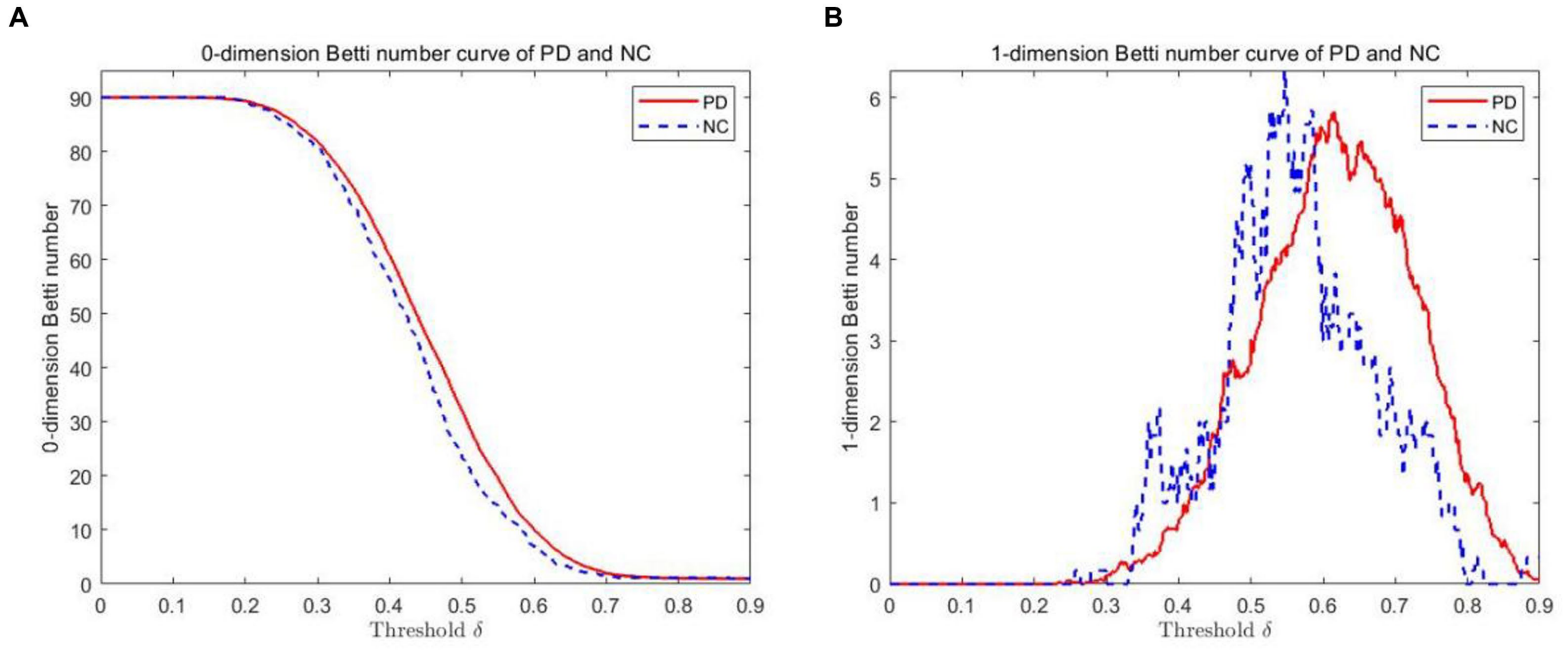
Betti number curves of PD and NC groups in 0 and 1 dimensions. **(A)** The 0-dimensional Betti number diagram. **(B)** The 1-dimensional Betti number diagram.

The VR complex is established under multiple scales. The 0-dimensional Betti number *β_0_* and the 1-dimensional Betti number *β_1_* under each scale are calculated using Javaplex software. Figure 3 plots show the 0-dimensional and 1-dimensional Betti number curves between PD and NC. As depicted from the 0-dimensional Betti number diagram (Figure 3A), with the increase of threshold δ, the number of connected branches of PD and NC gradually decrease from the initial 90. Until the threshold *δ* = 0.7, they tend to be consistent and gradually decreased to 1. But *β_0_* in PD is always greater than that in NC when *δ* ≤ 0.7. In the 1-dimensional Betti number curves (Figure 3B), the topological features was significantly different between the PD brain network and the NC brain network under different thresholds.

In Table 2, two sample Kolmogorov Smirnov (K-S) tests were also conducted from Figures 3A,B to compare whether there is a significant difference in distribution between the PD group and the NC group. From Table 2, it can be seen that the maximum absolute differences in the cumulative probability of Betti numbers in 0-dimension and 1-dimension are 0.049 and 0.082, respectively. Assuming a significance level of 0.05, as the probability *p*-values (both 0.000) are less than the significance level, it can be concluded that there is a significant difference in the Betti number curve between the PD group and the NC group, regardless of whether it is 0-dimensional or 1-dimensional. Therefore, by comparing the Betti numbers at all scales, the differences between these two groups can be detected.

**Table 2:** Two-sample K-S tests.

		0-dimension	1-dimension
Extreme difference	Absolute	0.049	0.082
	Positive	0.049	0.082
	Negative	−0.038	−0.058
Kolmogorov Sminov Z		3.451	5.819
Asymptotic significance (double tailed)		0.000	0.000

0-dimension and 1-dimension represents PD and NC samples of different dimensions separately.

**Figure 4:**
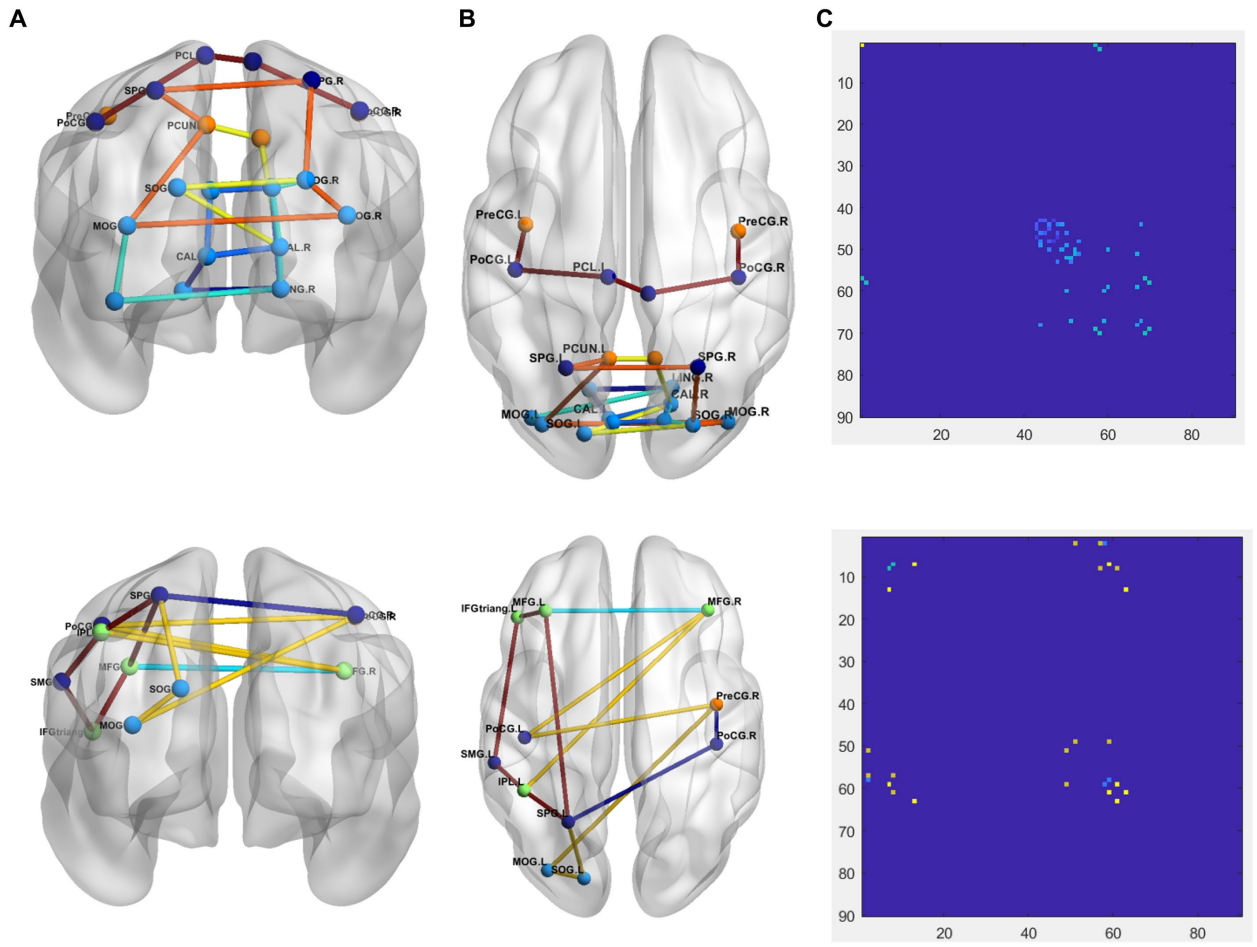
Comparison of the FCNC in the PD group and the NC group with *δ* = 0.4 in the Neurocon dataset. **(A)** Coronal view. **(B)** Sagittal view. **(C)** The relation matrix. The first row: the FCNC in the PD group. The second row: the FCNC in the NC group. Different colors are just for the purpose of easy observation.

To more intuitively show the difference between PD and NC groups, comparison of the 1-dimention FCNC in the PD group and the NC group with *δ* = 0.4 in the Neurocon dataset are shown in Figure 4. Among them, the coronal and sagittal views are shown in Figure 4A and Figure 4B respectively, and their corresponding relationship matrices are given in Figure 4C. Note that, the different colors in Figure 4 only represent different FCNC.

**Figure 5:**
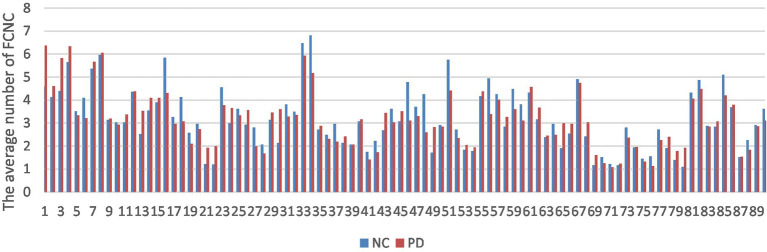
Comparison of the average number of FCNC between PD and NC groups in AAL 90 brain regions.

The number of FCNC in 90 brain regions was analyzed and a histogram was drawn in Figure 5. It can be seen intuitively, the average number of FCNC in PD and NC groups is significantly different in some brain regions. To further analyze these differences, the mean and standard deviation of the number of FCNC in each brain region were calculated for the PD and NC groups under thresholds of 0.7, 0.8, and 0.9 in Neurocon dataset, respectively. Then, two sample Mann Whitney *U* test and false discovery rate (FDR) correction were performed to detect differences between the two groups. The brain regions with statistical differences are shown in Table 3. In PD group, under threshold of 0.7, the number of FCNC involved was significantly differences and these brain regions include the Cuneus_R, Lingual_R, Fusiform_R and Heschl_R. In addition to the aforementioned brain regions, there are also significant differences in brain regions in the Frontal_Inf_Orb_R and Pallidum_R, when the threshold increases to 0.8 and 0.9 (*p* < 0.05 and FDR correction).

**Table 3:** Statistically significant differences in the numbers of FCNC in the involved brain regions.

Brain regions	Threshold	PD (*n* = 54) mean ± SD	NC (*n* = 31) mean ± SD	*P*-value	*q*-value (FDR-corrected)
Precentral_L	0.7	4.43 ± 3.611	3.03 ± 3.125	0.046*↓	0.0520
	0.8	6.07 ± 4.138	4.45 ± 3.075	0.039*↓	0.0520
	0.9	6.07 ± 4.138	4.52 ± 3.086	0.052	0.0520
Frontal_Inf_Orb_R	0.7	2.96 ± 2.747	3.97 ± 3.516	0.192	0.1920
	0.8	4.07 ± 3.392	5.74 ± 3.235	0.014*↓	0.0210*↓
	0.9	4.09 ± 3.388	5.81 ± 3.301	0.013*↓	0.0210*↓
Olfactory_R	0.7	0.63 ± 0.938	0.74 ± 0.999	0.672	0.6720
	0.8	1.81 ± 1.802	1.06 ± 1.181	0.059	0.0885
	0.9	1.91 ± 1.896	1.06 ± 1.181	0.043*↓	0.0885
Cuneus_R	0.7	2.15 ± 2.184	3.68 ± 2.774	0.008*↓	0.0210*↓
	0.8	2.91 ± 2.742	4.65 ± 3.431	0.018*↓	0.0210*↓
	0.9	2.93 ± 2.739	4.65 ± 3.431	0.021*↓	0.0210*↓
Lingual_R	0.7	1.81 ± 2.019	3.35 ± 3.251	0.035*↓	0.0350*↓
	0.8	2.33 ± 2.181	4.06 ± 3.255	0.010*↓	0.0150*↓
	0.9	2.35 ± 2.173	4.13 ± 3.274	0.008*↓	0.0150*↓
Occipital_Inf_R	0.7	0.98 ± 1.173	1.42 ± 1.205	0.041*↓	0.1230
	0.8	1.56 ± 1.690	1.77 ± 1.499	0.293	0.2930
	0.9	1.56 ± 1.690	1.77 ± 1.499	0.293	0.2930
Fusiform_R	0.7	2.35 ± 2.147	3.90 ± 2.508	0.003*↓	0.006*↓
	0.8	2.85 ± 2.227	4.68 ± 3.113	0.006*↓	0.006*↓
	0.9	2.85 ± 2.227	4.71 ± 3.090	0.005*↓	0.006*↓
Pallidum_R	0.7	0.43 ± 0.792	0.84 ± 1.293	0.085	0.0850
	0.8	0.81 ± 1.065	1.52 ± 1.480	0.007*↓	0.0150*↓
	0.9	0.83 ± 1.077	1.52 ± 1.480	0.010*↓	0.0150*↓
Heschl_R	0.7	1.19 ± 1.543	0.45 ± 0.675	0.013*↓	0.027*↓
	0.8	1.72 ± 1.857	1.06 ± 1.825	0.027*↓	0.027*↓
	0.9	1.72 ± 1.857	1.06 ± 1.825	0.027*↓	0.027*↓

SD, standard deviation; FDR, false discovery rate; ∗↓ denotes (*p* < 0.05).

We also analyzed the statistical differences in the number of FCNC with medium lengths in the Neurocon and PPMI datasets (Table 4). From Table 4, it can be seen that there is a significant difference between the PD group and the NC group for medium length of FCNC. Specifically, at thresholds of 0.8 and 0.9, there was a significant difference between the two groups in the Neurocon dataset (length = 8). When the thresholds are 0.7, 0.8, and 0.9, there was a significant difference between the two groups in the PPMI dataset (length = 9). All results were corrected through FDR correction. In these two datasets, at different thresholds, the mean of the PD group is always greater than that of the NC group.

**Table 4:** Statistically significant difference between the two groups in the number of medium length of FCNCs.

Datasets	Threshold value	PD (mean ± SD)	NC (mean ± SD)	*P*-value	*q*-value (FDR-corrected)
Neurocon	0.7	1.69 ± 1.286	1.30 ± 1.317	0.091	0.091
	0.8	2.33 ± 1.454	1.63 ± 1.377	0.017	0.043*↓
	0.9	2.330 ± 1.454	1.63 ± 1.377	0.029	0.043*↓
PPMI	0.7	1.14 ± 1.105	0.54 ± 0.977	0.004	0.008*↓
	0.8	1.36 ± 1.159	0.75 ± 1.113	0.008	0.008*↓
	0.9	1.38 ± 1.161	0.75 ± 1.113	0.007	0.008*↓

SD, standard deviation; FDR, false discovery rate; ∗↓ denotes (*p* < 0.05).

**Figure 6:**
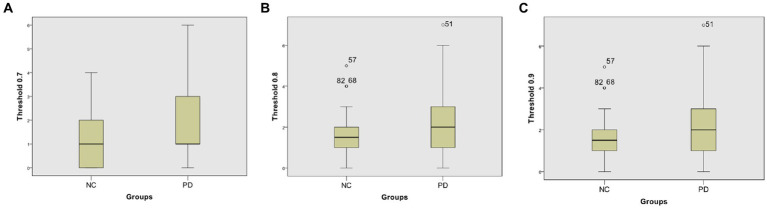
Box-plot comparison of the numbers of FCNC with length = 8. The numbers of FCNC with length = 8 when threshold = 0.7 **(A)**, 0.8 **(B)**, and 0.9 **(C)**, respectively.

At different thresholds, we can also visually see the significant differences between the two groups through the box-plot in the Neurocon dataset. From the box-plot Figure 6, it can be seen that when the threshold is 0.7, there is no significant difference in the median between the two groups. However, when the thresholds are increased to 0.8 and 0.9, the median of the PD group are significantly higher than that of the NC group. The above results show that there are significant differences in the characteristics of FCNC in some brain regions, and the medium length of FCNC in PD patients show significant changes.

According to the functional classification of the brain, Woo et al. (2014) divided the AAL brain regions into 7 sub networks. That is dorsal attention network (DAN), default mode network (DMN), the visual network (VSN), ventral attention network (VAN), limbic network (LBN), fronto parietal network (FPN) and somatomotor network (SMN) (Esposito et al., 2013; Baum et al., 2017).

By analyzing the FCNC, we found significant statistical difference in the Frontal_Inf_Orb, Cuneus, Lingual gyrus, Fusiform, Pallidum and Heschl areas. A similar result was obtained in previous studies regarding the function connectivity strength of the SMN and VAN (Caspers et al., 2021; Tsuboi et al., 2021) and our findings provide guidance for further studies on the pathogenesis of early PD. Interestingly, increases in FC within SMN (Pang et al., 2021) have been observed upon a dopaminergic challenge in PD patients. Both anterior central gyrus and transverse Nie gyrus belong to SMN. Caspers et al. (2021) pointed out that PD is accompanied by the loss of functional connection of SMN, whether within the network or in the interaction with other networks. The lesions of globus pallidus can have symptoms such as increased muscle tension, decreased movement and static tremor (such as Parkinson’s syndrome). Globus pallidus plays an important role in the regulation of motor function. It is not only the relay nucleus between the caudate putamen (CPU) and subthalamic nucleus (STN), but also integrates the inhibitory afferent from CPU and the excitatory afferent from STN, neocortex and thalamus, thus affecting the efferent signal of basal ganglia (Esposito et al., 2013). Globus pallidus stimulation can be used to improve the brain connectivity in order to treat advanced PD (Tsuboi et al., 2021).

## 5. Conclusion

This study applies persistent homology to the brain functional networks of PD. This work provides some new insights into the evolution of functional network in the progression of PD and may provide evidence for the study of preclinical biomarkers of PD. We observed that there are significant differences in the characteristics of FCNC in some brain regions, and the medium length of FCNC in PD patients show significant changes. Topological analysis based on rsfMRI data may provide comprehensive information about the changes of FCNC and may provide an alternative for clinical differential diagnosis.

## Data availability statement

Publicly available datasets were analyzed in this study. The Neurocon dataset can be found at: http://fcon_1000.projects.nitrc.org/indi/retro/parkinsons.html. The PPMI dataset can be found at: https://www.ppmi-info.org/.

## Author contributions

WZ, SX, and XZ designed the research study. DL performed the research. XT and YW wrote the manuscript. All authors contributed to editorial changes in the manuscript, read, and approved the final manuscript.

## Funding

This work was supported by the Natural Science Foundation of Shandong Province of China (ZR2020ZD25) and the Autonomous Innovation Team Foundation for “20 Items of the New University” of Jinan City (No. 202228087).

## Conflict of interest

The authors declare that the research was conducted in the absence of any commercial or financial relationships that could be construed as a potential conflict of interest.

## Publisher’s note

All claims expressed in this article are solely those of the authors and do not necessarily represent those of their affiliated organizations, or those of the publisher, the editors and the reviewers. Any product that may be evaluated in this article, or claim that may be made by its manufacturer, is not guaranteed or endorsed by the publisher.
